# Intrinsically flexible displays: key materials and devices

**DOI:** 10.1093/nsr/nwac090

**Published:** 2022-05-16

**Authors:** Zhiyuan Zhao, Kai Liu, Yanwei Liu, Yunlong Guo, Yunqi Liu

**Affiliations:** Key Laboratory of Organic Solids, Beijing National Laboratory for Molecular Sciences, Institute of Chemistry, Chinese Academy of Sciences, Beijing 100190, China; School of Chemistry and Chemical Engineering, University of Chinese Academy of Sciences, Beijing 100049, China; Key Laboratory of Organic Solids, Beijing National Laboratory for Molecular Sciences, Institute of Chemistry, Chinese Academy of Sciences, Beijing 100190, China; School of Chemistry and Chemical Engineering, University of Chinese Academy of Sciences, Beijing 100049, China; Key Laboratory of Organic Solids, Beijing National Laboratory for Molecular Sciences, Institute of Chemistry, Chinese Academy of Sciences, Beijing 100190, China; School of Chemistry and Chemical Engineering, University of Chinese Academy of Sciences, Beijing 100049, China; Key Laboratory of Organic Solids, Beijing National Laboratory for Molecular Sciences, Institute of Chemistry, Chinese Academy of Sciences, Beijing 100190, China; School of Chemistry and Chemical Engineering, University of Chinese Academy of Sciences, Beijing 100049, China; Key Laboratory of Organic Solids, Beijing National Laboratory for Molecular Sciences, Institute of Chemistry, Chinese Academy of Sciences, Beijing 100190, China; School of Chemistry and Chemical Engineering, University of Chinese Academy of Sciences, Beijing 100049, China

**Keywords:** intrinsically flexible display, intrinsically flexible electrode, organic semiconductor, transistor driver, electroluminescent device

## Abstract

Continuous progress in flexible electronics is bringing more convenience and comfort to human lives. In this field, interconnection and novel display applications are acknowledged as important future directions. However, it is a huge scientific and technical challenge to develop intrinsically flexible displays due to the limited size and shape of the display panel. To address this conundrum, it is crucial to develop intrinsically flexible electrode materials, semiconductor materials and dielectric materials, as well as the relevant flexible transistor drivers and display panels. In this review, we focus on the recent progress in this field from seven aspects: background and concept, intrinsically flexible electrode materials, intrinsically flexible organic semiconductors and dielectric materials for organic thin film transistors (OTFTs), intrinsically flexible organic emissive semiconductors for electroluminescent devices, and OTFT-driven electroluminescent devices for intrinsically flexible displays. Finally, some suggestions and prospects for the future development of intrinsically flexible displays are proposed.

## Teaser

This review introduces the concept, key materials and device structure design of intrinsically flexible displays, and proposes some potential solutions to current concerns and future development.

## INTRODUCTION

**Figure 1.**
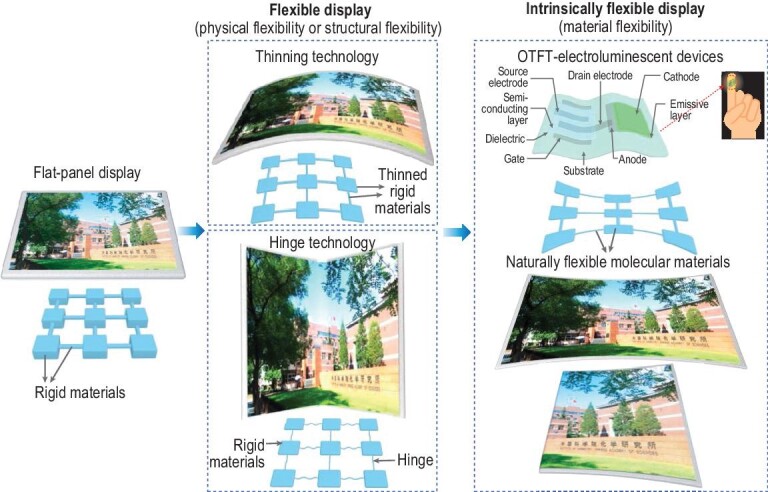
Schematic diagrams showing the development process of a display.

Over the last two decades, the continuous development of display technologies has shown great potential to make human lives more vibrant and comfortable. Display technologies have progressed from thick electronic tube technology to the thin liquid crystal (LC) strategy and the current flexible organic light-emitting diode (OLED) technology. Compared with conventional rigid displays, flexible displays offer several unique characteristics, such as being lightweight and having ultrathin structure, low energy consumption and high-mechanical deformation-tolerance capacity. Thus, they have garnered considerable attention from industry and academia. For example, a foldable display can endure a large-scale bending angle of 180º with a bending radius below 5 mm or even below 1 mm. However, based on current thinning technology and hinge design (Fig. 1), the reported flexible OLED-based displays and thin-film transistor (TFT)-driven systems cannot endure <1 mm bending radius and multidimensional bending. They are primarily limited by the rigid and brittle nature of the existing components in the displays, such as silicon-based TFT.

Technically, there are different routes for the realization of flexibility (Fig. 1). (i) Physical flexibility: any rigid material that is extremely thin or has a very small diameter can be flexible. (ii) Structural flexibility: for example, the wire-connecting fractal and spring configuration can provide macroscopic flexibility for the rigid chip. (iii) Intrinsic flexibility: the materials (such as polymers and carbon materials) in a device have a flexible and stretchable nature. The combination of physical and structural flexibility has facilitated the development of hinge technology in folding-screen phones. However, the long-term mechanical stability of such phones is still a considerable challenge. Usually, the flexibility is quantified by the bending radius (R). A decreased R resulted in the improved flexibility of materials. Different from flexible and stretchable devices, intrinsically flexible displays should simultaneously satisfy three critical requirements: large elastic deformation, small bending radius (<0.5 mm), and high stretching strain (>25%), which decides whether they can be subsequently conformed, folded or rolled (Fig. 1). With these three conditions, intrinsically flexible displays can change the perception of information that frequently appears in almost all aspects of our lives.

Advancements in material and manufacturing technologies are driving the development of intrinsically flexible electronics. It has been extensively reported that the use of organic materials with a flexible and stretchable nature is key to realizing intrinsically flexible displays [1,2]. In this review, great importance is attached to the key materials for intrinsically flexible organic thin-film transistors (OTFTs) and electroluminescent devices. Specifically, we focus on the following aspects: intrinsically flexible electrode materials, organic semiconductors (OSCs) and dielectric materials for OTFTs, intrinsically flexible organic emissive semiconductors (OESCs) for electroluminescent devices, and OTFT-driven electroluminescent devices for intrinsically flexible displays. Lastly, existing and future challenges and opportunities with regard to intrinsically stretchable OTFT-driven displays are presented.

## INTRINSICALLY FLEXIBLE ELECTRODE MATERIALS

Electrodes that sustain stable conductivity under mechanical deformations are of crucial significance for stretchable and wearable electronic devices. Conventional metal oxides, such as indium tin oxide (ITO) and aluminum-doped zinc oxide (AZO), have been frequently employed as electrode materials for flexible electronic devices. However, the high electrical conductivity of these metal oxide electrodes requires compact homogeneous film structures, which is detrimental for mechanical compliance and stretchability [3,4]. Fortunately, since the emergence of nanotechnology, some stretchable nanoscale electrode materials such as carbon nanotubes (CNTs), graphene, metal nanowires (MNWs), conducting polymers (CPs) and their hybrids have been proposed. Such conducting nanomaterials can be used—individually and also in combination with other polymers—to obtain an intrinsically flexible electrode material [5]. Here, we will provide a detailed summary of intrinsically flexible electrode materials based on material category, manufacturing method and structural design.

### CNTs

CNTs have some distinctive performance characteristics, such as excellent electrical conductivity (≈10^4^ S/cm), superior mechanical robustness, outstanding thermal stability, low sheet resistance (<100 Ω) and high optical transparency (>90%) [6]. A CNT electrode can be obtained by firstly dispersing raw nanoparticles in water or alcohol solvent and then depositing these nanoparticles onto various stretchable substrates via drop-casting, spray-casting or spin-coating methods.

**Figure 2.**
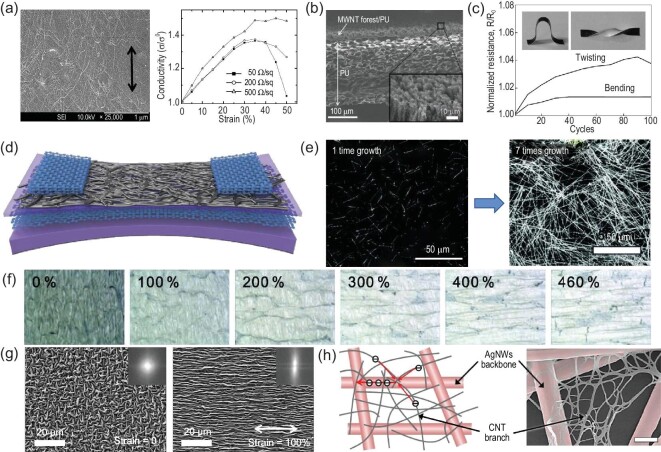
(a) Scanning electron microscopy (SEM) image of the SWCNT-polymer composite electrodes under 100% tensile strain (the arrow shows the stretching direction) [9]. Copyright 2011, WILEY-VCH. (b) SEM image of the sheet cross section for the rubber-like MWCNT forest/PU composites. The inset shows a high-resolution image of the black forest side [10]. Copyright 2010, WILEY-VCH. (c) Normalized resistance (R/R_0_) of the MWCNT forest/PU composites as a function of repeated bending and twisting cycles. The insets represent the sample shape induced by bending (left) and twisting (right) deformation [10]. Copyright 2010, WILEY-VCH. (d) Diagram showing the stretchable transistor using the multilayer graphene/graphene scrolls as electrodes under stretching [13]. Copyright 2017, The American Association for the Advancement of Science. (e) SEM image of AgNWs prepared via the successive multistep growth method after one-time and seven-time growth, respectively [19]. Copyright 2012, WILEY-VCH. (f) Microscopic surface morphology of the stretchable, very long AgNW percolation network (VAgNPN) electrodes under stretching strain from 0% to 460% [19]. Copyright 2012, WILEY-VCH. (g) SEM images of the PEDOT: PSS/g-PDMS electrode film without (left) and with 100% (right) stretching strain. Insets show the Fourier transform of the SEM images [28]. Copyright 2019, American Chemical Society. (h) Schematic representation (left) and SEM image (right) of the hierarchical multiscale AgNW/CNT hybrid nanocomposites. The inset scale bar is 300 nm [30]. Copyright 2014, WILEY-VCH.

CNT film has been extensively used as the intrinsically flexible gate, source and drain electrodes for stretchable electronics. Lipomi *et al*. reported a transparent and conducting spray-deposited single-walled CNT (SWCNT) electrode with relatively stable electrical conductivity against tensile strain [7]. The formation of spring-like structures in the nanotubes could afford SWCNT electrodes high tensile strain exceeding 150% and high conductivity approaching 2200 S/cm. However, the rough and irregular surface morphology of CNT electrodes obtained by the solution deposition significantly degrades the interfacial contact between electrodes and the semiconductor layer. To this end, blending CNTs with elastomeric binders can reduce the surface roughness of CNT electrodes and improve the interfacial quality between electrodes and semiconductors [8]. Yu *et al*. proposed a simple strategy to prepare stretchable, transparent SWCNT-polymer composite electrodes by blending SWCNTs with poly (tert-butyl acrylate) (PtBA) (Fig. 2a) [9]. The resultant conducting composites demonstrated low-sheet resistance, high transparency, superior flexibility and low surface roughness, which were attributed to the formation of interpenetrating networks in the polymer matrix. Apart from SWCNTs, multiwalled CNTs (MWCNTs) have also attracted considerable research interest as an intrinsically flexible electrode material. Shin *et al*. fabricated a stretchable, conducting composite through the direct infiltration of MWCNTs forests in the polyurethane (PU) solution (Fig. 2b) [10]. The rubber-like forest/PU composites showed highly reversible stress-strain behavior, with stable electrical conductivity (Fig. 2c).

### Graphene

Graphene is a one-atom-thick two-dimensional (2D) layer of sp^2^-bonded carbon allotrope. High-quality graphene film can show low sheet resistance (100−1000 Ω sq^–1^) and high transparency (>90%) [11]. However, graphene has high in-plane stiffness (>340 N/m) and Young's modulus (>0.5 TPa), and the strong internal C−C networks are detrimental because of the energy dissipation induced by the applied strain, due to which graphene film is easily fractured at low stretching strain (<6%) [12]. It is reported that the strength and stiffness of graphene can be reduced by stacking it into multiple layers. The impedance changes of the bi- or tri-layer graphene were 13 times lower than that of the mono-layer graphene under 30% strain. In view of this strategy, Liu *et al*. developed multilayer graphene/graphene scrolls (MGGs), which could retain 65% of the initial electrical conductivity under 100% stretching strain (Fig. 2d) [13]. The improved strain tolerance was ascribed to the formation of delocalized microcracks in graphene film when subjected to stretching strain, which improved the endurance of the applied strains by deviating the out-of-plane strain modes and delaying the strain localization [14]. It is universally acknowledged that graphene oxide (GO) has simple synthesis and cost-effective solution processing [15,16]. Zhou *et al*. fabricated a highly stretchable, conductive composite electrode consisting of 2D titanium carbide (Ti_3_C_3_T_x_) MXene and reduced graphene oxide (RGO) for high-performance stretchable supercapacitors [17]. The MXene/RGO composite electrode combined the excellent mechanical and electrochemical properties of Ti_3_C_3_T_x_ with the outstanding electrical conductivity of RGO. These composite electrodes exhibited a high capacitance of ∼49 mF/cm^3^ and superb electrochemical stability under large cyclic uniaxial (300%) and biaxial (200% × 200%) strains.

### MNW electrodes

MNW electrodes have extraordinary electrical characteristics but weak transparency and mechanical robustness. Although reducing the thickness of metal films can partially improve their flexibility and transparency, it still cannot meet the requirements of intrinsically flexible electrode materials. Actually, metal (such as Ag, Cu and Ni) nanowires (NWs) can be employed as intrinsically flexible electrode materials because of their high thermal/electrical conductivity and mechanical compliance. The high aspect ratio of MNWs is beneficial for retaining good electrical contact between nanowires under stretching strain.

Among MNWs, Ag nanowires (AgNWs) are the most frequently used intrinsically flexible electrode material due to their low sheet resistance (≈9 Ω sq^–1^), high light transmittance (≈89%), excellent bending performance and much lower cost than ITO [18]. In general, AgNWs are prepared through the reduction of Ag nitrate, with poly (vinyl pyrrolidone) (PVP) as the solvent and ethylene glycol as the catalyst. However, the resulting conductive films usually show high sheet resistance due to large wire–wire junction resistance. Lee *et al*. synthesized long AgNWs (Fig. 2e) with an ultrahigh tensile strain above 460% (Fig. 2f) and a low sheet resistance [19]. Furthermore, they embedded AgNWs into elastomers to improve the conductivity and stretchability. However, the direct blending of AgNWs with elastomers cannot form uniform conductive networks due to phase separation, high viscosity, etc. Recently, Choi *et al*. developed a highly stretchable, conductive and biocompatible Ag−Au nanocomposite by dispersing ultralong Ag−Au NWs into the poly(styrene-butadiene-styrene) (SBS) elastomer [20]. The Ag−Au nanocomposites showed a maximum conductivity exceeding 72 600 S/cm due to the high aspect ratio and high-density percolation networks of Ag−Au NWs. The microstructures induced by phase separation in the Ag−Au nanocomposites yielded a maximum stretchability of 840%. Meanwhile, the epitaxial deposition of the Ag sheath on the AgNW's surface efficiently reduced the oxidation and leaching of Ag ions.

In addition to AgNWs, the Cu-, Ni- and Au-based NWs have also been utilized as intrinsically flexible electrodes. Cu (*ρ* = 16 nΩ m) has a slightly lower conductivity than Ag (*ρ* = 17 nΩ m), but it is nearly 1000 times more abundant and 100 times cheaper than Ag. However, Cu is easily oxidized, and it is reddish orange in color. To overcome this problem, Rathmell *et al*. synthesized cupronickel NWs by coating transparent CuNWs with nickel shells [21]. The oxidation resistance of cupronickel NWs is ∼1622 times and 135 times higher than that of CuNWs and AgNWs, respectively. Moreover, when the weight ratio of Cu:Ni was 2:1, the cupronickel NWs showed a neutral gray color, which satisfied the requirements of flexible displays and electrochromic windows. As mentioned above, Ni has a higher oxidation resistance than Cu. Li *et al*. prepared highly stretchable NiNWs with excellent conductivity by directly drop-casting unidirectional NiNWs on a PU matrix [22]. The prepared conductive film could endure 300% stretching strain. Further, the conductivity of the film gradually increased with increasing strain due to the improved electrical contacts between the twisted NWs under tension. It is a considerable challenge to prepare the AuNWs with a similar size as AgNWs. Lyons *et al*. fabricated highly conductive and transparent AuNWs with an average NW diameter of 47 nm and a mean thickness of 35−700 nm [23]. Obviously, there existed a large difference in the thickness of AuNWs, which severely impacted their electrical conductivity. The conductivity was ∼3 kΩ. Although the flexibility and stretchability of AuNW has never been investigated, it is still expected to be suitable for stretchable electronics due to its ultrahigh aspect ratio.

### CPs

CPs, especially poly(3,4-ethylenedioxythiophene): polystyrene sulfonate (PEDOT: PSS), have been extensively used as flexible electrodes, owing to their excellent film uniformity and cost-effectiveness. The PEDOT:PSS film can be easily patterned through various strategies, such as inkjet printing, photolithography, selective wetting-based patterning and screen printing. [24]. Just by the spin-coating method, the film can exhibit quite a low sheet resistance (≈46 Ω sq^–1^) and a high transmittance (≈82%) [25]. However, the pristine PEDOT: PSS film is susceptible to humidity, and is vulnerable to strain (<5%) [26]. Fortunately, the flexibility and stretchability of the PEDOT: PSS film can be improved by the incorporation of elastomers or surfactants, structural engineering, and the formation of micro/nanostructures [27,28].

Usually, additives can partly increase the flexibility of the PEDOT: PSS film, but they inevitably cause a decrease of conductivity. Wang *et al*. fabricated a highly stretchable conductive polymer on the polystyrene-block-poly(ethylene-ran-butylene)-block-polystyrene (SEBS) substrate by introducing an ionic additive into the PEDOT: PSS solution. The prepared polymer film exhibited record-high stretchability (>800%) and conductivity (3100 S/cm). Even under 600% stretching strain, the polymer film could maintain a conductivity over 100 S/cm [27]. However, the surface energy of PEDOT: PSS (hydrophilic) films is incompatible with stretchable substrates such as poly(dimethylsiloxane) (PDMS) (hydrophobic). To enhance the interfacial adhesion and mechanical matching between PEDOT: PSS and PDMS, Li *et al*. fabricated the PEDOT: PSS film on a biaxially pre-strained poly (methacrylic acid) (PMAAc)-grafted (methyl methacrylate) (PMMA) substrate (Fig. 2g) [28]. The formation of hydrogen bonds enhanced the interfacial contact between the PEDOT: PSS film and PDMS substrate, and the large folds and wrinkles improved the stretchability of the conductive film. The conductive film exhibited a low resistance of 90 Ω/sq. Moreover, no cracks were observed on the film under 100% strain for 10 00 cycles. In addition to the PEDOT-based polymers, some other conducting polymers such as polyaniline, polypyrrole and polythiophene could also be used as electrode materials in intrinsically flexible electronic devices [29].

### Hybrid electrodes

It is well known that each material has its own advantages and disadvantages in practical applications. CNT electrodes usually indicate high stretchability and good compatibility with flexible substrates, but they have inferior conductivity, low transparency and high material cost, and present difficulties with regard to scalable fabrication. Moreover, CNT electrodes prepared by solution deposition show rough film surface, which severely degrades the interfacial contact between electrodes and semiconductors. Graphene electrodes indicate high optical transparency, low sheet resistance and large-scale manufacturing, but they typically have weak strain resistance (>6%). Metallic NWs typically demonstrate high conductivity, superb corrosion stability and oversimplified fabrication, but they suffer from poor mechanical stability, low breakdown voltage and poor adhesion to plastic substrates. CPs have low sheet resistance, good film uniformity, low cost and simple film patterning, but they are easily damaged by humidity and stretching strain (>5%). Consequently, only one material is unsuitable as the intrinsically flexible electrode. How to combine the advantages of different types of electrode materials and remove the disadvantages of one electrode material has been a research focus for intrinsically flexible electronic devices.

Lee *et al*. prepared a hierarchical multiscale AgNW/CNT hybrid conductive nanocomposite to overcome this obstacle [30]. In the hybrid nanocomposite structure, an efficient multiscale electron transport path was formed through the current-carrying backbone region of AgNWs and the local percolation network of CNTs (Fig. 2h). The resultant hybrid nanocomposites showed high stretchability (>460%), high optical transparency (80%−93%) and relatively stable conductivity under large-scale continuous bending (>10 00 times), twisting (>540º) and folding (∼0º, complete folding). There are several types of hybrid electrodes, including graphene−AgNW hybrid nanostructures [31], AgNW/PEDOT:PSS [32], AgNW/SWCNT [33], Ag nanoparticle/RGO [15], AgNW/RGO [34] and RGO/CNT/AgNW [35], which exhibit excellent conductivity and stretchability.

## INTRINSICALLY FLEXIBLE OSCs FOR OTFTs

**Figure 3.**
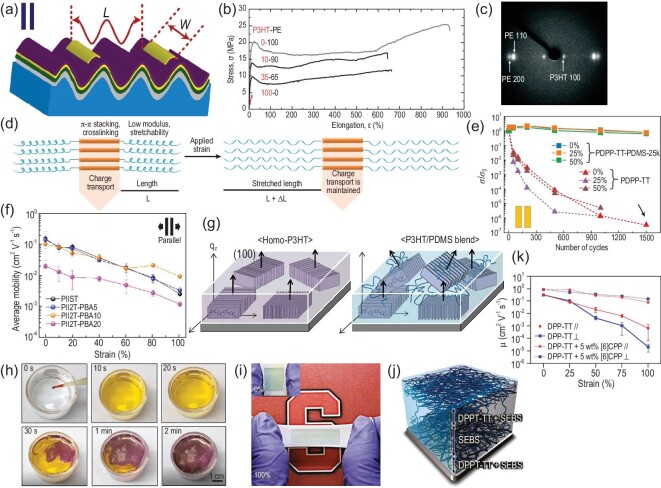
(a) Stretchable thin-film transistors fabricated on the topographic substrate with source/drain electrodes parallel to the topographic direction [43]. Copyright 2013, Elsevier B.V. (b) Stress-elongation profile of the diblock copolymer with different weight ratios of P3HT and polyethylene (PE) [47]. Copyright 2007, WILEY-VCH. (c) The wide-angle X-ray diffraction patterns of a 35−65 P3HT-PE film under 600% stretching strain, which demonstrates a high degree of uniaxial order and crystallinity [47]. Copyright 2007, WILEY-VCH. (d) Visualization of the phase segregation concept. Charge carriers are transported through the stacked inner poly[2,5-(2-octyldodecyl)-3,6-diketopyrrolopyrrole-alt-5,5-(2,5-di(thien-2-yl)thieno [3,2-b]thiophene)] (DPP-TT) blocks and are retained even when stretching strain is applied on the elastic PDMS blocks [49]. Copyright 2020, WILEY-VCH. (e) The DC conductance of the neat PDPP-TT and PDPP-TT-PDMS polymer under 0∼50 tensile strain and multiple stretching cycles (0∼1500) parallel to the charge-transporting direction [49]. Copyright 2020, WILEY-VCH. (f) Average carrier mobility of the PII2T-PBA block polymers under stretching strain from 0% to 100% parallel to charge-transporting direction [53]. Copyright 2017, American Chemical Society. (g) Schematic illustration of the distribution of P3HT NWs in homo-P3HT (left) and P3HT NW/PDMS blend films (right) on Si wafer [59]. Copyright 2015, WILEY-VCH. (h) Fabrication process of the P3HT nanofilm on the water surface [61]. Copyright 2020, The American Association for the Advancement of Science. (i) Three-dimensional (3D) schematic illustrating the dispersion of poly(2,5-bis(2-octyldodecyl)-3,6-di(thiophen-2-yl)diketopyrrolo[3,4-c]pyrrole-1,4-dione-alt-thieno[3,2-b]thiophen) (DPPT-TT) polymer in the SEBS matrix for the CONPHINE film [62]. Copyright 2017, The American Association for the Advancement of Science. (j) Morphology of the CONPHINE film on a PDMS substrate under 0% and 100% stretching strains [62]. Copyright 2017, The American Association for the Advancement of Science. (k) Mobility of fully stretchable transistors with neat DPP-TT and DPP-TT/5 wt% CPP blends as the semiconducting materials, under 0%∼100% stretching strains, parallel and perpendicular to the charge-transporting direction [64]. Copyright 2019, WILEY-VCH.

As a critical component of electronic devices, OSCs, especially polymer semiconductors (PSCs), demonstrate great potential in intrinsically flexible electronics. Conventional brittle polymers can be afforded mechanical flexibility through various geometrical and structural strain-engineering strategies, such as pre-strain elastomers [36], net configurations [37], wrinkles [38], serpentine [39], kirigami structural configuration [40], buckling [41] and wavy structures [42], but they cannot really be called intrinsically flexible OSCs. For example, Wu *et al*. adopted a strain-relief mechanism to construct OTFTs on the prefabricated topographic elastomeric substrate (Fig. 3a). The as-fabricated devices retained high mobility and on/off current ratios with slight strain-relief characteristics under 12% tensile strain [43]. However, these strategies have complicated the fabrication of high-density and large-area integrated circuits. Meanwhile, due to the unchanged brittle and stiff nature of PSCs, the stretchability of polymers could be generally improved below 20% strain, which does not satisfy the requirement of intrinsically flexible displays.

The crystallinity and molecular packing of OSCs are responsible for improved electrical performance, but they are detrimental to mechanical compliance and stretchability. The existing approaches to prepare intrinsically flexible OSCs are primarily divided into the following categories [44,45]: structurally designing polymer chains through the incorporation of conjugation-break spacers (CBSs) and flexible chain segments, controlling the molecular weight and regioregularity of conjugated polymers, and blending with elastomer polymers or molecular additives.

### Polymer backbone design

Based on the monomeric level, the types and the sequence of monomers as well as the overall polymer design can affect the mechanical and electrical properties of PSCs. For instance, O’Connor *et al*. systematically analyzed the structure–property relationship of two polythiophenes: poly(3-hexylthiophene) (P3HT) and poly(2,5-bis(3-alkylthiophene-2-yl) thieno[3,2-*b*] thiophene) (pBTTT) [46]. It was observed that the carrier mobility of pBTTT (μ_h_ = 0.34 cm^2^ V^−1^ s^−1^) was higher than that of P3HT (μ_h_ = 0.06 cm^2^ V^−1^ s^−1^), whereas the elongation at the point of breaking of pBTTT (<2.5%) was much lower than that of P3HT (>150%). These results indicated that the long-range order was beneficial for high mobility but caused the stiffness and brittleness of polythiophene films. Therefore, the highly ordered lamellar structure and high crystallinity are detrimental to the mechanical stretchability, which requires an amorphous morphology.

To address this conundrum, Müller *et al*. synthesized a tough, semiconducting polyethylene-poly(3-hexylthiophene) (P3HT-PE) diblock copolymer by introducing insulating flexible polyethylene (PE) sequences within P3HT polymer [47]. Based on copolymers with 90 wt% of insulating PE moiety, the prepared OTFTs demonstrated a mobility of 0.02 cm^2^ V^–1^ s^–1^ and an on/off current ratio of 10^5^. Additionally, the diblock copolymers showed a supramolecular stretchability with an elongation at breaking point exceeding 600% and a tensile strength of ∼70 MPa (Fig. 3b), which was due to the high degree of uniaxial order and crystallinity of the diblock copolymer under 600% tensile strain (Fig. 3c). Using thermoplastic elastomers, such as polystyrene-b-polyisoprene-b-polystyrene (SIS) and poly(styrene-butadiene-styrene), as flexible moiety, Peng *et al*. successfully synthesized P3HT-PMA-P3HT triblock copolymers (TBCs), which could self-assemble into a well-ordered nanofibrillar structure under annealing conditions [48]. The copolymer films displayed a high elongation at breaking point up to 140% and a carrier mobility of 9 × 10^–4^ cm^2^ V^–1^ s^–1^.

As mentioned above, block copolymers are composed of two or more types of thermodynamically incompatible polymers, which facilitates the formation of micro-phase segregation with improved mechanical and electrical performance. Ditte *et al*. synthesized TBCs using PDMS as the elastomeric unit to endcap a donor–acceptor (D−A) conjugated polymer (PDPP-TT) [49]. Based on molecular engineering, the PDPP-TT blocks were seen as the extended conjugated system functioning as the cross-linker to PDMS by π−π interaction (Fig. 3d). The prepared TBCs with 65 wt% PDMS content demonstrated a high carrier mobility reaching 0.1 cm^2^ V^–1^ s^–1^ and could endure a high tensile strain of 50% for 1500 cycles without noticeable degradation in the electrical properties (Fig. 3e). 2,6-pyridine dicarboxamide (PDCA), with two amide groups, can provide intermolecular hydrogen bonds in the flexible backbone. Oh *et al*. attempted to incorporate PDCA moieties within the semiconducting polymers to promote the formation of non-covalent cross-linking of polymer backbone [50]. These non-covalent cross-linking bonds exhibited excellent dynamic stability and were able to undergo continuous breakage-recovery processes under applied and released tensile strain. After releasing from 100% stretching strain and 100 stretching cycles, the prepared PDCA-containing PSCs maintained close to their original mobility, ∼1 cm^2^ V^–1^ s^–1^. Significantly, even if the device was severely damaged, the carrier mobility could still recover to almost the original value using thermal and solvent annealing. Such effective polymer backbone design strategies provide useful insights into the development of high mobility, large stretchability and even outstanding self-healing capability in intrinsically flexible OSCs.

### Polymer side chain design

Alkyl side chains are traditionally considered to impede the transport of carriers due to weak intermolecular interactions and localized aggregates in conjugated polymers [51]. Nevertheless, recent reports indicated that the D−A conjugated polymer with different long-branched side chains showed high carrier mobilities regardless of the weak crystalline nature [52]. Wen *et al*. synthesized a series of isoindigo-bithiophene (II2T)-based conjugated polymers with varying contents (0, 5, 10, 20 and 100%) of poly (butyl acrylate) (PBA) side chains [53]. The incorporation of PBA side chains endowed the conjugated polymers with better processability, higher mechanical ductility and lower elastic modulus, and the elastic modulus gradually decreased with the increasing incorporation of PBA segment. These conjugated polymers exhibited a carrier mobility of 0.06−0.08 cm^2^ V^–1^ s^–1^ and an on/off current ratio of ∼10^5^. Moreover, at the PBA content of 10%, the conjugated polymers maintained a high mobility of 0.08 cm^2^ V^–1^ s^–1^ under 60% strain over 400 stretching-releasing cycles (Fig. 3f). Therefore, a clear understanding of the crystalline and amorphous domains in PSCs can provide guidelines for synthesizing high-mobility intrinsically flexible OSCs.

### Controlling molecular weight and regioregularity

Molecular weight (Mn) and regioregularity (RR) have a certain impact on the morphology and electronic properties of PSC films. In general, PSCs are synthesized through two routes: step-growth condensation reaction and chain-growth polymerization reaction. The former route is suitable for synthesizing high-mobility D−A copolymers. However, the reaction is extremely sensitive to various parameters such as solvent, temperature, concentration, monomer reactivity and purity, which causes profound batch-to-batch discrepancies in the Mn and dispersity (Đ) of samples, even under meticulous experimental operation. By contrast, the latter route can perfectly control the Mn and Đ of PSCs, but it is only applicable when synthesizing specific conjugated polymers such as P3HT [45].

Kline *et al*. investigated the effects of Mn and Đ on the electrical performance and mechanical compliance of polymer P3HT [54]. The results revealed that the mobility of P3HT film was strongly dependent on its Mn, and the mobility of OTFTs increased from 1.7 × 10^−6^ to 9.4 × 10^−6^ cm^2^ V^–1^ s^–1^ when the Mn increased from 3.2 kDa to 36.5 kDa. Further, Rodriquez *et al*. found that low-Mn P3HT samples (15 kDa) were easily fractured, whereas for high-Mn (<25 kDa) samples, the molecular chain entanglements easily concentrated the stress to a few chains [55]. Improved fracture resistance by increasing the Mn is explained by Koch *et al*. [56]. The polymer film with lower Mn (<25 kg/mol) tended to form chain-extended structures, whereas that with higher Mn tended to form fringed-micelle microstructures. Ultimately, the polymer film with high Mn (110 kg/mol) exhibited a uniaxially oriented structure under high stretching strain, which resulted in a high elongation at break exceeding 300% and a stress at breaking point of 24 MPa.

Apart from Mn, the RR is another parameter that impacts the crystallinity of polymer films and their mechanical and electrical properties. For the conjugated polymer chain structure, the head-to-head and tail-to-tail coupling configurations are relevant to the twisting of the polymer backbone, whereas the head-to-tail coupling configuration is relevant to the ordered interchain packing. Therefore, the RR of PSCs can be regulated by adjusting the monomer connection mode. Kim *et al*. synthesized a sequence of conjugated polymers, P3HT, with varied RR from 64% to 98% by controlling the incorporation of head-to-head coupled dimer [57]. When the Mn and Đ were almost similar, the P3HT films with high RR (98%) exhibited a high carrier mobility but high stiffness and brittleness. This was because the increase of RR facilitated easy crystallization and suppressed the evolution of chain orientation in the polymer films. In contrast, the film with RR of 13% exhibited a significant reduction in the tensile modulus from 287 MPa to 13 MPa and also demonstrated a one-order-magnitude increase in the elongation at break.

It could be concluded that high Mn is responsible for forming fringed-micelle microstructures, while the low R is responsible for suppressing crystallization and facilitating polymer orientation. Therefore, increasing the Mn and decreasing the R are beneficial for enhancing the mechanical compliance of PSCs, which is a general concept and is also adapted to other high-performance D−A copolymers.

### Polymer blends and composites

It has been reported that by realizing homogeneous micro-scale phase separation between conjugated polymers and elastomers, their conjugated polymer/elastomer hybrids can simultaneously retain efficient charge transport pathways and high stretchability. In addition, the hybrids have some unique characteristics, such as low material cost, tunable activation energy, adjustable solidification kinetics and high environmental stability [58]. The P3HT/PDMS blends are the most extensively studied intrinsically flexible OSCs. The formed P3HT NWs in elastomer matrix can maintain original percolated networks and excellent electrical performance against stretching due to the random rotation and alignment (Fig. 3g) [59]. However, preparing highly semiconducting P3HT/PDMS blends requires a proper pretreatment of P3HT solution, optimum blending ratio and suitable processing techniques. Choi *et al*. prepared high-performance stretchable P3HT/PDMS blends [60]. The formation of the ordering π−π stacked P3HT fibrillar networks in a PDMS matrix improved the carrier mobility (60 times higher than neat P3HT film) and stretchability (>100%).

Additionally, SEBS is another common elastomer to blend with conjugated polymers. Four critical procedures are essential for obtaining high-performance intrinsically flexible OSCs: *in-situ* phase separation of P3HT nanofibrils in SEBS matrix, the assembly of nanofibrils into wide bundles, the formation of bundle networks and the stable bondage of nanofibrils on the SEBS surface. Guan *et al*. prepared high-performance stretchable SEBS/P3HT semiconducting nanofilms based on a novel air/water interfacial assembling strategy (Fig. 3h) [61]. The SEBS/P3HT blend solution was gently dropped into water, and quickly spread on the water surface due to the Marangoni effect. The blended nanofilms were formed via the evaporation of toluene solvent. The OTFTs based on the blend nanofilms, with 65 wt% P3HT, exhibited a high hole mobility of 8.6 cm^2^ V^–1^ s^–1^. When subjected to 50% stretching strain, the mobility slightly decreased to 6.8 cm^2^ V^–1^ s^–1^ and returned to 8.2 cm^2^ V^–1^ s^–1^ upon releasing the strain.

Recently, Bao's group proposed a nanoconfinement-effect concept to fabricate high-performance intrinsically flexible OSCs [62]. High-quality semiconductor film morphology could be achieved based on a conjugated-polymer/elastomer phase-separation-induced elasticity (CONPHINE) strategy (Fig. 3i). Because of the nanoconfinement structure, the chain dynamics of conjugated polymers were greatly improved, thereby reducing the elastic modulus and delaying the onset of crack formation under stretching strain. All the CONPHINE films showed no noticeable cracks under 100% tensile strain (Fig. 3j), and could retain a high carrier mobility of 1 cm^2^ V^–1^ s^–1^ against repeated biaxial stretching-releasing cycles at 100% strain. Further, they employed a solution-shearing strategy to fabricate high-mobility CONPHINE films (μ > 1.6 cm^2^ V^−1^ s^−1^) without sacrificing strain tolerance. The increased mobility was attributed to the improved multiscale and short-range π−π ordering and alignment of conjugated polymers [63].

In addition to the above strategies, Mun *et al*. established the concept of single ‘universal’ molecular additives in conjugated polymers [64,65]. The molecular additives not only improved the dynamic behavior and short-range ordered aggregates of conjugated polymers but also retained fiber-like morphology. They prepared polymer composites by introducing two types of molecular additives, namely cycloparaphenylenes (CPPs) and 2,3,5,6-tetrafluoro-7,7,8,8-tetra-cyanoquinodimethane (F4-TCNQ), in the diketopyrrolopyrrole (DPP)-based polymers. Compared to the pristine DPP-based polymer film, the prepared composite film exhibited higher carrier mobility (2.66-fold increase for DPP-based polymers/CPPs and 1.5-fold increase for DPP-based polymer/F4-TCNQ) and higher strain tolerance (Fig. 3k). Moreover, the addition of such molecular additives was beneficial for reducing the contact resistance and improving environmental stability. These findings provide a simple and promising methodology for realizing high-performance intrinsically flexible OSCs.

## INTRINSICALLY FLEXIBLE DIELECTRIC MATERIALS FOR OTFTs

Unlike flexible electrode and semiconductor materials, there are few options for flexible dielectric materials. The gate dielectric is close to the semiconductor layer, and significantly affects the electrical performance of devices [66]. However, conventional dielectric materials such as PMMA, polyphenyleneoxide (PPO), poly (α-methylstyrene) (PαMS) and poly (vinylalcohol) (PVA) cannot satisfy the requirements of intrinsically flexible dielectric materials due to their brittle and stiff nature.

Presently, common elastomeric dielectric materials include PU (dielectric constant of 3.5−4.3) with elongation at break up to 800%, PDMS (dielectric constant of 3−5) with elongation at break up to 500%, and SEBS (dielectric constant of 2.5−3.5) with elongation at break exceeding 900%. The intrinsically flexible dielectric materials should possess high mechanical compliance, excellent environmental and chemical stability, easy solution processing, etc. Meanwhile, increasing the dielectric constant of dielectric materials is of crucial significance for enabling low power consumption of flexible electronic devices. To satisfy these requirements, it is essential to perform suitable modifications or cross-linking on elastomeric materials. For example, Rao *et al*. prepared metal-salt PDMS based on the metal-ligand coordination by introducing metal ions (Fe^2+^ and Zn^2+^) and bipyridine to the PDMS matrix [67]. The metal-salt cross-linked PDMS not only improved the dielectric constant and stretching strain but also exhibited an autonomous self-healing capacity. Du *et al*. reported that the introduction of conducting polymer nanowires (CPNWs) to the PDMS matrix could significantly increase the dielectric constant by <4 times compared with neat PDMS [68]. To improve the solvent-resistance behavior of SEBS dielectrics, Wang *et al*. employed azide-cross-linking chemistry to prepare azide-cross-linked SEBS based on the reaction of azide groups with C−H groups [69].

## INTRINSICALLY FLEXIBLE ORGANIC EMISSIVE SEMICONDUCTORS FOR ELECTROLUMINESCENT DEVICES

Flexible OESCs are divided into three categories: general emissive materials with low stretchability (<20%), soft matrix-induced high-stretchability emissive materials (>30%) and intrinsically stretchable block polymers. As mentioned above, organic materials have a flexible and elastic nature, facilitating the development of bendable OLED-based displays. Moreover, the deformation-dependent optoelectronic properties of these materials also act as a prototype, which reveals the charge carrier transport model [70] and aids the development of deformation-induced functional materials [71].

**Figure 4.**
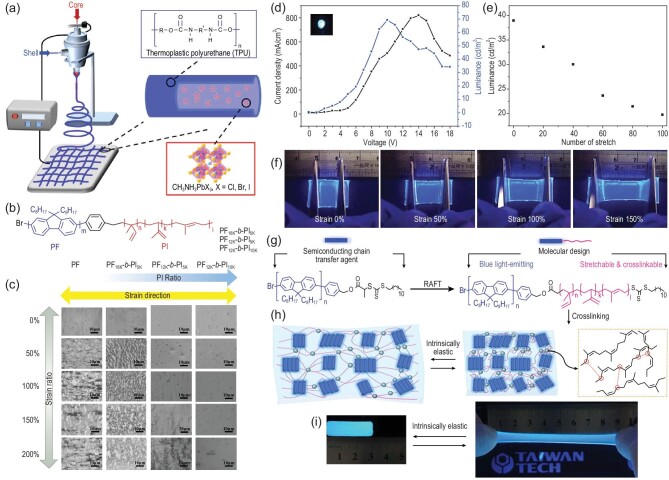
(a) Schematic of the coaxial electrospinning core-shell polyurethane (TPU)/perovskite luminous nanofibers [76]. Copyright 2019, American Chemical Society. (b) Molecular structure of the PF-b-PI rod-coil block copolymer with different rod/coil ratio configurations [79]. Copyright 2022, John Wiley and Sons. (c) Photographic images of the copolymer films under a strain of 0%, 50%, 100%, 150% and 200% [79]. Copyright 2022, John Wiley and Sons. (d) I−V−L characteristic curves of the stretchable PF_12K_-b-PI_5K_ LEDs [79]. Copyright 2022, John Wiley and Sons. (e) Luminance-stretching cycle characteristics of a device under different cycles at a constant strain of 20% [79]. Copyright 2022, John Wiley and Sons. (f) Photographic images of the PF_12K_-b-PI_5K_ LEDs under ultraviolet light and a strain of 0%, 50%, 100% and 150% [79]. Copyright 2022, John Wiley and Sons. (g) Molecular structure of the stretchable PF-b-PI copolymer [78]. Copyright 2020, American Chemical Society. (h) Schematic illustration of the deformable polymer with a PF-b-PI_1.8_ matrix and cross-linker 1, 9-nonanedithiol (DT). Blue-rectangular block, pink fibril and gray sphere denote the PF rod, PI coil and the cross-linked point, respectively [78]. Copyright 2020, American Chemical Society. (i) Photographic images of the cross-linked stretchable PF-b-PI_1.8_ film at a strain of 0% and 300% [78]. Copyright 2020, American Chemical Society.

Limited by weak intermolecular interactions, these organic materials usually show low stretching strain, below 20%. With the composite assembly strategy and soft matrix doping, many luminescent materials have higher strain tolerance and self-healing capacities [72,73]. The same strategy was also adapted to perovskites and quantum dot materials, and the stretchability of these materials has been realized through assembly configurations such as gel films [74] and nanofibers [75]. The doping of flexible connection components affords perovskites and quantum dot materials stretchable luminescence, which makes it possible to prepare stretchable LEDs [76] (Fig. 4a). However, for most existing luminescent polymers, it is difficult to achieve charge transfer efficiency while maintaining a certain stretchability through conventional assembly and flexible matrix doping strategies. The weak coupling strength design of emissive polymers usually causes low backbone conjugation and intermolecular secondary assembly. Consequently, the emissive polymers typically exhibit a severe decrease in mobility under plastic deformation and elastomer matrix doping. Kasparek *et al*. demonstrated that >20% insulator doping significantly affected the charge transfer capacity of emissive polymers [77]. This means that the blending of insulating polymer into emissive polymer would inevitably sacrifice the charge transfer ability. Considering the weak intramolecular conjugation of emissive polymers, secondary assembly engineering is a potential strategy for molecular design.

In addition to the doping strategy, copolymerization is another effective approach to designing intrinsically flexible OESCs. Rigid rods connected with elastomeric coils facilitate polymer rigid island-soft bridge stacking, ensuring improved stretchability and optoelectronic properties [78]. Jao *et al*. developed stretchable LEDs using a new conjugated rod–coil block copolymer, poly [2,7-(9,9-dioctylfluorene)]-block-poly (isoprene) (PF-b-PI) [79]. At a suitable rod/coil ratio, the LED could work well even under 140% stretching strain (Fig. 4b−f). The elastomeric spacer lent good stretchability to the emissive polymer, but sacrificed charge transfer efficiency. A similar strategy was used by Au-Duong *et al*., where a rigid poly (9,9-di-n-octyl-2,7-fluorene) (PFO)-conjugated rod and soft poly (isoprene) (PI) coils were connected to obtain PF-b-(PI)x [78]. The obtained PF-b-PI_1.8_ could sustain 150% strain over 1000 stretching cycles without losing its photoluminescence quantum yield (PLQY) (Fig. 4g−i).

Perovskite and quantum dot materials show obvious advantages in intrinsically flexible OESCs due to their tunable structural and photoelectric characteristics as well as excellent charge transfer capacity. By combining appropriate semiconductors and additives, the stretchability of low-dimensional perovskite and quantum dot materials can be further improved. Although the emissive polymers have low crystallinity and high aggregation, they usually exhibit low crack-onset strain, which is because these emissive polymers have not enough freed volume to dissipate external strain. According to the design concept of intrinsically flexible OESCs, the modulus and tensile properties of emissive polymers can be improved by introducing secondary force fragments. The existing block copolymerization strategy can effectively improve the tensile properties of luminescent polymers, but there is an obvious trade-off between mechanical and electro-optical properties. Compared with traditional polymers and luminescent materials, through-space charge transfer (TSCT) delayed fluorescent materials developed by Wang *et al*. [80] and Ren *et al*. [81] demonstrated high PLQY, programmable D–A spatial arrangement and excellent performance in traditional polymer light-emitting diodes (PLEDs). By combining elastic block fragments, mechanical and optoelectronic properties can be simultaneously improved in the next generation of intrinsically flexible OESCs.

## INTRINSICALLY FLEXIBLE ELECTROLUMINESCENT DEVICES

Intrinsically flexible electroluminescent devices with good conformability and elastic response can widen the application of display platforms in the next-generation electronics industry, in areas such as stretchable smart displays [82], visualized electronic skin [83] and biomedical imaging [84]. Versatile soft materials and stretchable transparent electrodes are combined with emissive semiconductors to achieve intrinsically flexible electroluminescent devices. Several researchers have demonstrated this by employing structural and material engineering. Next, we provide a detailed introduction to intrinsically flexible OLEDs, organic light-emitting cells (OLECs), LC displays and other electroluminescent devices.

### Intrinsically flexible OLEDs

**Figure 5.**
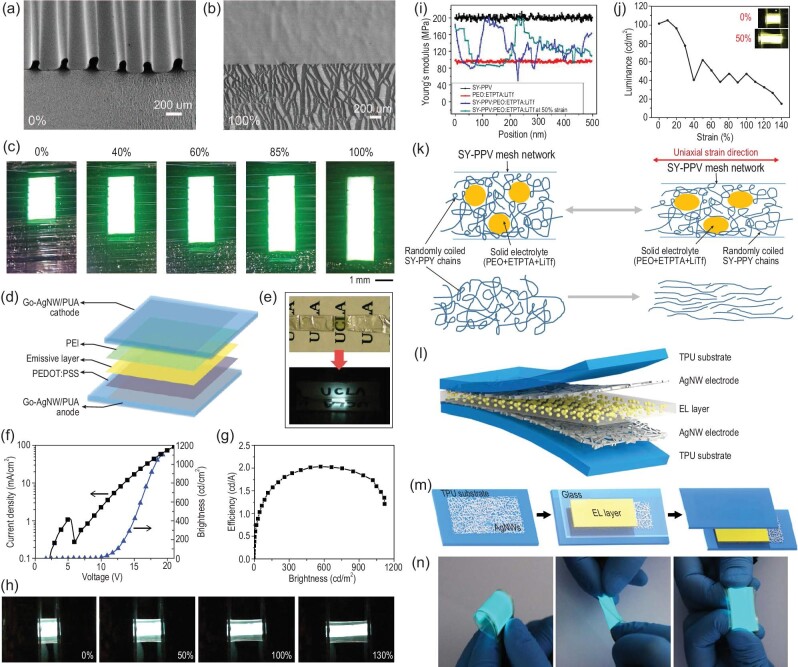
SEM images of the stretchable OLEDs at (a) 0% strain and (b) 100% strain. (c) Photographic images of the stretchable OLEDs at varying strain values from 0% to 100% [85]. Copyright 2019, American Chemical Society. (d) Device structure of the fully stretchable OLED using GO-AgNWs/PUA composite electrode as counter electrodes [86]. Copyright 2014, American Chemical Society. (e) Photographic images of a semi-transparent unlit stretchable PLED (top) and a stretchable PLED illuminated at a voltage of 13 V (bottom) [86]. Copyright 2014, American Chemical Society. (f) Current-voltage curve and (g) luminescence-current efficiency curve of the stretchable PLED [86]. Copyright 2014, American Chemical Society. (h) Photographic images of a stretchable PLED at different strains (initial emissive area of 3 mm × 4 mm with a driven voltage of 14 V) [86]. Copyright 2014, American Chemical Society. (i) Cross-sectional Young's modulus curves of different samples obtained from peak force quantitative nanomechanical mapping. The mixture of SY-PPV and polymer electrolyte forms an effective nano-interpenetrating network to achieve tensile properties [90]. Copyright 2016, American Chemical Society. (j) Brightness-strain characteristic curve of polymer light-emitting electrochemical cell (PLEC) [90]. Copyright 2016, American Chemical Society. (k) Schematic of the nano-interpenetrating networks under different strains. The solid electrolyte dissipates the external stress, thereby preserving the general phase morphology of SY-PPV [90]. Copyright 2016, American Chemical Society. (l) Structure of the alternating current electroluminescent (ACEL) device [96]. Copyright 2018, American Chemical Society. (m) Fabrication route of the ACEL device [96]. Copyright 2018, American Chemical Society. (n) Photographic images of the ACEL device powered by a commercial electroluminescence (EL) driver under bent, twisted and stretched states [96]. Copyright 2018, American Chemical Society.

Amorphous stacking could provide a certain level of stretchability for most luminescent polymers and transport materials. Firstly, creating stretchable electrodes is the key to developing intrinsically flexible OLEDs. White *et al*. fabricated an intrinsically flexible OLED by binding ultrathin OLED film onto pre-strained elastomer [1]. This structural strategy facilitated truly stretchable characteristics under 100% tensile strain and 10 μm bending radius. The buckling structure and directional randomness can boost the light-extraction efficiency without any side effects on the spectral characteristics. Consequently, the current and power efficiency of these devices are improved, indicating their potential in high-performance intrinsically flexible OLEDs. Recently, Yin *et al*. used roller-assisted adhesion imprinting to manufacture a high-throughput, large-area (length >10 cm) intrinsically flexible OLED with regular buckling structure (Fig. 5a−c) [85]. The device exhibited a high current efficiency of 70 cd A^−1^ under 70% strain and only 5% variation in the current efficiency over 15 00 stretching-releasing cycles under 20% deformation.

Although OLEDs based on the pre-wrinkling and ultrathin strategy are intrinsically flexible, these devices are incompatible with display panel design. In addition to intrinsically flexible electrode materials and intrinsically flexible OSCs, a stretchable charge carrier injection layer is also crucial for creating intrinsically stretchable OLEDs. The main obstacle of the intrinsically flexible OLED are the elastic stretchability and stability of the interface interlayer, emissive layer and electrode. Intrinsically flexible transparent electrode materials with stable roughness and conduction properties under tensile deformation are indispensable for creating high-performance intrinsically flexible OLEDs. Liang *et al*. developed a fully stretchable white PLED with GO−AgNWs−polyurethane acrylate (PUA) composite electrode (Fig. 5d−h) [86]. The percolation network of GO-modified AgNWs reduced the inter-nanowire contact resistance. The *in-situ* substrate polymerization of siliconized urethane acrylate oligomer (UAO) and inter-band unit of ethoxylated bisphenol A dimethacrylate (EBA) effectively enhanced the stretchability of electrodes. Consequently, a high strain above 100% was realized without losing electrical conductivity. The PLED could survive at 40% strain over 100 stretching-releasing cycles, and retained 130% linear stretching strain limitation at room temperature. Large-strain deformation inevitably influences the film morphology and interface contact, finally degrading the electroluminescent properties of stretchable PLEDs.

Rigid-structured perovskite film can be used to fabricate intrinsically flexible perovskite light-emitting diodes (IFPerLEDs) with auxiliary flexible additives such as poly (ethylene oxide) (PEO) and PVP. The polymer matrix serves as an elastic microscale connector, which affords the perovskites higher stretchability. Recently, Jiang *et al*. reported a stretchable touch-responsive perovskite LED with AgNW/PU composite electrodes [87]. The composite emissive layer was formed by adding 12.5 wt% of PEO and 2.25 wt% of PVP to the CsPbBr_3_ perovskite. A thin PVP layer was inserted between the emissive layer and PEDOT: PSS-PVP bottom electrode to serve as an electron-injection barrier. Additionally, a 100-μm-thick poly (ethylene terephthalate) (PET) spacer was inserted between the top electrode and the emissive layer. This stretchable touch-responsive PerLED could sustain a 30% uniaxial strain with a 24% luminescence drop-off. Surprisingly, the stretchable PerLED showed touch on-off responsivity over 300 cycles and low operation voltage (turn-on voltage of 2 V with a brightness of 380.5 cd m^−2^ at 7.5 V). Limited by the energy-level requirement and work-function alignment, the development of intrinsically flexible OLEDs lags behind that of other intrinsically flexible LEDs.

### Intrinsically flexible OLECs

Common PLEDs simplify the multilayer configuration into three positive-interlayer-negative (P−I−N) layers sandwiched between the electrodes. However, the thickness-sensitive positive–negative (P−N) charge injection interlayers are detrimental to the preparation and stable operation of stretchable devices. Recently, an OLEC has been developed using an elastomer binder-phosphor-ionic conducting medium composite with single layer configuration, which effectively circumvented the multilayered structure and sophisticated energy alignment of general OLEDs [88]. The composite active layer enables a built-in P−N or P−I−N junction within the two-electrode system, thereby realizing an efficient charge injection, transport and emission process. Owing to the high thickness tolerance of active layers, reduced material interfaces and barrier-less injection, the OLEC device exhibits immense potential in high-performance stretchable electronics [89]. Electrochemical doping of the semiconductor effectively removes the injection barrier, thereby discarding the unnecessary interfacial dipole layer and enabling a high-power efficiency. The diverse electroluminescent components, binder components and ionic components endow the active layer with more free volume, allowing microscale phase separation or interpenetrating networks to be obtained and the desired stretching properties to be achieved.

Improving the performance of intrinsically flexible PLECs is dependent on the transparent electrode and luminous layers. The first intrinsically flexible PLEC was reported by Yu *et al*. in 2011 based on the composite electrodes of SWCNTs and PtBA [9]. The *in-situ* photopolymerization of a liquid monomer penetrating the porous SWCNT coating on glass effectively enhanced the surface smoothness and the adhesion of SWCNTs to substrate. Benefitting from the interpenetrating network structure and the shape memory ability of a polymer matrix, the composite electrode exhibited low sheet resistance and high transparency, and could sustain 50% strain without sacrificing sheet resistance. The roll lamination process was used to sandwich the polyfluorene copolymer (PFB), PEO−dynamic mechanical analysis (DMA) and Lithium trifluoromethane sulfonate (LiTf) emissive layer between the two composite electrodes to form a PLEC device. The prepared device achieved a maximum efficiency of ∼1.24 cd A^−1^ at 200 cd m^−2^ and could bear up to 45% strain without damaging the EL properties. Interestingly, the electrolyte with low glass transition temperature (Tg) is an effective soft additive for easing the deformation of emissive polymer as it operates as a solid plasticizer in the semi-crystalline regions of the blends. This means that the soft electrolyte dissipates the stress, thereby facilitating the application of traditional emissive polymers in intrinsically flexible PLECs. In addition, a composite emissive layer formulated with soluble alkyloxy phenyl substituted poly(1,4-phenylene vinylene) (SY-PPV), exoxylated trimethylolpropanetriacrylate (ETPTA), PEO and LiTf was used to fabricate stretchable PLECs. The optimized device reached a peak current efficiency of ∼11.4 cd A^−1^ (corresponding to an external quantum efficiency near 4.0%) and could sustain 120% strain. Notably, the cross-linked network formed by ETPTA and the PEO additive effectively enhanced the stretchability of the emissive layer. The improved stretchability of the emissive composite was explained in detail by Gao *et al*. [90]. Benefitting from the relatively low modulus of the solid electrolyte, an interpenetrating polymer network was formed when an ionically conductive medium containing poly (ethylene oxide), exoxylated trimethylolpropanetriacrylate and lithium trifluoromethanesulfonate was admixed with the super yellow (SY-PPV). Peak force quantitative nanomechanical mapping revealed that the composite film exhibited an obvious phase separation with a high local Young's modulus, while the isolated islands were much softer with a low Young's modulus (as shown in Fig. 5i−k). The soft porous solid electrolyte functioned as the strain dissipation under external stimuli, thereby ensuring the unaffected accumulation of conjugated polymers. The dichroism of optical absorption and the anisotropic fluorescence spectra confirmed that the continuous SY-PPV mesh in the blended film largely retained the randomness of SY-PPV chains under stress due to the formed microphase separation. Accordingly, the PLEC device could be stretched up to 140% strain without EL polarization. Compared with the general PLED based on pristine SY-PPV, the PLEC device with composite exhibited more competitive current–voltage (I−V) characteristics [90]. However, the luminescence of the stretchable PLEC significantly declined under stretching stress (>50% reduction under 40% stretching stress). Extensive experiments must be further conducted on the charge transfer characteristics, PLQY and I−V properties of the emissive layer and the corresponding device under stretching stress to elucidate the performance deterioration. Furthermore, it should be noted that the mixing of elastomer generally degrades the charge-transport ability of conjugated polymers. In most cases, the elastomer is inevitably inserted into the region of the conjugated polymer, which increases the charge transfer resistance between the conjugated polymer chains. This significantly influences the EL performance of the stretchable device. Therefore, the assembling of conjugated polymers should be carefully regulated to guarantee the nanoconfinement (or matrix-nanowire inclusion) morphology of the blended elastomer-conjugated polymer instead of the general domain phase separation stacking.

In addition to conjugated polymers, perovskite materials are another potential candidate for fabricating PerLECs [91]. The adjustable component composition and morphology are beneficial for designing and selecting perovskite materials for PerLECs. Yu *et al*. reported a perovskite light-emitting electrochemical cell (PerLEC) based on a PEO−MAPbBr_3_ composite emitter sandwiched between a PEO–PEDOT: PSS composite anode and a liquid metal (EGaIn) top cathode. The PEO additive not only enhanced the stretchability of commercial PEDOT: PSS but also acted as a soft matrix to bear the rigid micrometer-sized MAPbBr_3_ crystal grains. Consequently, the synergy between the organic and inorganic components facilitated the high brightness (15 960 cd m^−2^ at 8.5 V) and low operation voltage (turn-on voltage of 2.4 V) of the PerLEC device. Further, a reversible stretching of 40% strain for 100 cycles was realized.

The above results indicate that LECs have unique advantages in stretchable displays, and their simple structure, and the stretchable design freedom of the luminescent layer, enhance tensile strength. To improve the stability and stretchability of devices, choosing appropriate emissive materials and linkers is a research focus for the future.

### Intrinsically flexible LC displays

The LC driver is generally employed as a low-cost driver technology in display panels and recently developed curved displays, with the aid of blank or columnar spacers [92,93]. To surmount the weak mechanical performance of the normal wall-stabilized LCs, different kinds of gelators have been developed to maintain the traditional orderliness and fluidity of LC through a 3D network structure, which simultaneously improved the external stimulus response and mechanical characteristics of LC films. Tong *et al*. developed an electroresponsive, stretchable LC device with a deformable gel network [94]. The stretchable LC device consisted of a super-strong LC gel network (5CB/POSS-G1-BOC) and transparent conductive electrodes that were prepared by embedding AgNWs in a PU matrix. A ‘spiral dissipate energy’ model was proposed to describe the energy dissipation. The levorotatory nanofibers formed from the self-assembly of the gelators transformed into rod-like nanofibers to dissipate energy induced by external stimuli, thereby enabling excellent light-transmission control of the LC device during successive 45% stretching. These results suggest the potential of LC gel devices in wearable displays and electronic skin.

### Other electroluminescent devices

Alternating current electroluminescent (ACEL) devices are generally formed by a phosphor-embedded emissive layer sandwiched between transparent electrodes. Doping of the rigid phosphor microparticles into a high-k elastomeric matrix can effectively reduce the operating voltage and improve the mechanical compliance of the active layer [95]. Kong's group developed a series of high-performance, low-voltage stretchable ACEL devices based on surface-modified phosphors or high-k elastomeric matrix doping (Fig. 5l−n) [96]. These strategies effectively enhanced the EL performance of the ACEL devices under low driving voltage. Interestingly, screen printing was used for the scalable fabrication of a fully screen-printed, multicolor and stretchable electroluminescent display [97]. These devices demonstrated stable EL properties under stretching. To avoid the limited stretchability of the

devices due to the previously reported stretchable transparent electrode, Wang *et al*. fabricated a stretchable ACEL device using ionic conductors [95]. The ionic conductor showed high stretching strain up to 700%. Notably, the ACEL device exhibited increased emission intensity at stretching strain below 280%, and maintained 70% of the initial intensity at 700% strain. All these results indicate the potential of stretchable ACEL devices with regard to stretchable lighting and volumetric 3D displays.

### Intrinsically flexible OTFT-driven electroluminescent devices

The combination of intrinsically stretchable OTFTs makes it possible to create an intrinsically stretchable display matrix circuit, which offers new opportunities for wearable displays as well as direct visual interaction and feedback.

Someya *et al*. constructed a rubber-like stretchable OLED active-matrix display based on an elastic printable conductor [98]. The uniformly dispersed SWCNTs within the fluorinated rubber enabled stretchable electrodes for OTFTs. The display demonstrated 30%−50% stretchability without any electrical damage. However, the rigid structure of the evaporated OLED deteriorated the elasticity of this panel.

**Figure 6.**
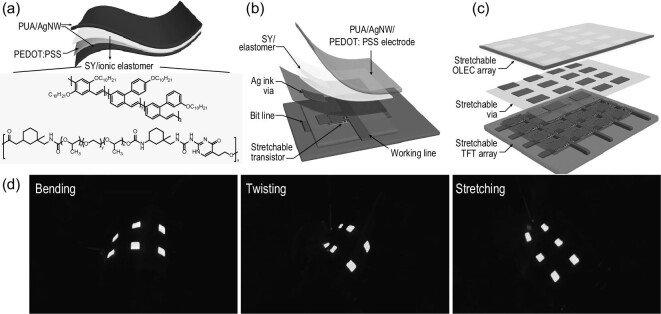
(a) Device structure of the stretchable light-emitting electrochemical cell, where the molecular structures of super yellow and stretchable ion-conducting polymer are highlighted [99]. Copyright 2020, Springer Nature. (b) 3D structural layout of a single active-matrix organic light-emitting electrochemical cell (AMOLEC) pixel [99]. Copyright 2020, Springer Nature. (c) 3D layout scheme of the vertical integrated active matrix and PLEC array [99]. Copyright 2020, Springer Nature. (d) Photographic images of the AMOLEC skin display pixels subjected to bending, twisting and stretching [99]. Copyright 2020, Springer Nature.

To overcome the rigidity of traditional OLEDs, Bao *et al*. combined intrinsically stretchable PLEC with the intrinsically stretchable OTFTs, and demonstrated the first fully stretchable active-matrix organic light-emitting electrochemical cell array (Fig. 6a−d) [99]. A chemically orthogonal and stretchable elastomer was used to fabricate the active matrix by bottom-up vertical integration of the intrinsically stretchable OTFT array with stretchable PLEC array. Interfacial adhesion regulation strategies, including cross-linking and the elastomeric interlayer, could improve the stability of the multilayer device. The resulting array could be readily twisted and stretched, and it could sustain multiple stretching cycles at 30% strain. Notably, all-solution processing would enable scalable manufacturing of future large-scale skin-display devices.

## CONCLUSION AND FUTURE OUTLOOK

Based on the advantages of OSCs and OTFTs, OTFT-based displays are expected to become the basic units of intrinsically flexible displays. Although major breakthroughs have been achieved in intrinsically flexible materials and technologies, many issues still remain unresolved in terms of the practical realization of intrinsically flexible displays.

How to create intrinsically flexible OSCs with high carrier mobility is a main focus of attention. Blending elastomers and conjugated polymers is a common strategy for preparing stretchable polymer semiconductors. However, only P3HT and DPP-based conjugated polymers were exploited in stretchable polymer semiconductors. N-type and bipolar stretchable polymer semiconductors have never been created. The blending mechanism, and conditions such as surface energy and molecular weight, need to be clarified. Meanwhile, research on the unified structure–property relationship of the structure-strain-electro-optical properties of intrinsically flexible OSCs (including transport materials and luminescent materials) is still in a nascent stage, and further investigations and new design concepts need to be developed for the stretchable electro-optical properties of materials.

Substrate materials can directly affect the bending radius of intrinsically flexible displays. Their selection is strongly dependent on the dielectric materials and fabrication procedure of devices. The difference in the elastic modulus between dielectric materials and substrate materials easily induces the Poisson effect and causes weak interfacial contact during multiple stretching-releasing cycles. Therefore, it is essential to ensure the similar stress relaxation and surface energy of a dielectric layer and substrate. In addition, most of the reported stretchable electrodes, such as CNTs and AgNWs, have high surface roughness, which is detrimental to the interfacial contact between electrodes and the active layer. Blending with elastomeric binders and constructing layer-heterojunction electrodes are promising strategies for overcoming this issue.

The performance of OTFT-driven electroluminescent devices can be improved by morphology regulation (crystalline state, orientation) and interface improvement. Therefore, the development of solution-processing technology and interface engineering is responsible for the improved optoelectrical performances. However, high-resolution patterns on multilevel scales are still a research focus and a challenge. Moreover, the integration of multiple functional devices often requires a reasonable circuit design and a comprehensive performance measurement. Many issues need to be addressed, such as the slippage between functional layers, device consistency, stability and repeatability.

Overall, opportunities and challenges coexist, and we strongly believe that with the cooperative efforts of academia and industry, a significant breakthrough can be attained. In the near future, intrinsically flexible displays will substantially change our lifestyle.
